# Three‐dimensional assessment of interfractional cervical and uterine motions using daily magnetic resonance images to determine margins and timing of replanning

**DOI:** 10.1002/acm2.14073

**Published:** 2023-06-15

**Authors:** Yukako Kishigami, Mitsuhiro Nakamura, Megumi Nakao, Hiroyuki Okamoto, Ayaka Takahashi, Hiroshi Igaki

**Affiliations:** ^1^ Department of Advanced Medical Physics Graduate School of Medicine Kyoto University Kyoto Japan; ^2^ Department of Biomedical Engineering and Intelligence Graduate School of Medicine Kyoto University Kyoto Japan; ^3^ Radiation Safety and Quality Assurance Division National Cancer Center Hospital Tokyo Japan; ^4^ Department of Radiation Oncology National Cancer Center Hospital Tokyo Japan

**Keywords:** cervical cancer, daily MR images, interfractional variations, margin, replanning

## Abstract

**Purpose:**

This study was conducted to determine the margins and timing of replanning by assessing the daily interfractional cervical and uterine motions using magnetic resonance (MR) images.

**Methods:**

Eleven patients with cervical cancer, who underwent intensity‐modulated radiotherapy (IMRT) in 23–25 fractions, were considered in this study. The daily and reference MR images were converted into three‐dimensional (3D) shape models. Patient‐specific anisotropic margins were calculated from the proximal 95% of vertices located outside the surface of the reference model. Population‐based margins were defined as the 90th percentile values of the patient‐specific margins. The expanded volume of interest (expVOI) for the cervix and uterus was generated by expanding the reference model based on the population‐based margin to calculate the coverage for daily deformable mesh models. For comparison, expVOI_conv_ was generated using conventional margins: right (R), left (L), anterior (A), posterior (P), superior (S), and inferior (I) were (5, 5, 15, 15, 10, 10) and (10, 10, 20, 20, 15, 15) mm for the cervix and uterus, respectively. Subsequently, a replanning scenario was developed based on the cervical volume change. ExpVOI_ini_ and expVOI_replan_ were generated before and after replanning, respectively.

**Results:**

Population‐based margins were (R, L, A, P, S, I) of (7, 7, 11, 6, 11, 8) and (14, 13, 27, 19, 15, 21) mm for the cervix and uterus, respectively. The timing of replanning was found to be the 16^th^ fraction, and the volume of expVOI_replan_ decreased by >30% compared to that of expVOI_ini_. However, margins cannot be reduced to ensure equivalent coverage after replanning.

**Conclusion:**

We determined the margins and timing of replanning through detailed daily analysis. The margins of the cervix were smaller than conventional margins in some directions, while the margins of the uterus were larger in almost all directions. A margin equivalent to that at the initial planning was required for replanning.

## 1 INTRODUCTION

In recent years, intensity‐modulated radiotherapy (IMRT) has been widely used to treat cervical cancer as it significantly reduces acute gastrointestinal and genitourinary (GU) toxicities and chronic GU toxicity in patients compared to three‐dimensional (3D) conformal radiotherapy.^1^, ^2^ However, interfractional variations, including tumor regression and target positional change, are the most important issues that must be addressed owing to the risks of tumor under‐dosing and/or normal tissue over‐dosing.^3^, ^4^, ^5^, ^6^, ^7^, ^8^, ^9^, ^10^, ^11^, ^12^, ^13^, ^14^, ^15^, ^16^

Although many researchers have studied such variations, there has been a lack of daily observation, individual assessment distinguishing the cervix and uterus with high‐contrast medical images, and 3D evaluations.^8^, ^9^, ^10^, ^11^, ^12^, ^13^, ^14^, ^15^, ^16^ Several researchers have reported margins for cervical cancer without daily observations or individual assessments, and all of them used two‐dimensional (2D) evaluations.^8^, ^9^, ^10^, ^11^, ^12^ Bondar et al. designed a 3D model to predict the shape and position of the cervix and uterus. However, they did not perform daily imaging and did not assess the cervix or uterus separately.^13^ Although some studies revealed the effectiveness of replanning against tumor regression, they were still not based on daily MR images.^14^, ^15^, ^16^ Online adaptation systems have emerged in recent years. Although the effectiveness of target coverage with a smaller margin and OAR sparing has been proven,^17^, ^18^ several human and time resources are required. Moreover, the ownership rate of online adaptation systems is low worldwide. Online adaptive radiotherapy with library plans is one approach for improving OAR sparing.^19^, ^20^ However, such a system is not commercially available. Therefore, the appropriate margin and timing of replanning are important issues that need to be addressed for cervical radiotherapy. To address the issues, a precise assessment of interfractional movement is required.

This study introduced two completely different techniques. First, a 3D evaluation approach was developed to assess organ motions by applying a shape model to express variations in their shape.^21^, ^22^ Second, daily MR images acquired from an MR‐guided radiotherapy (MRgRT) system were used. The MRgRT system provided daily high‐contrast images, thereby allowing independent motion analysis of the cervix and uterus. This study was aimed at determining the margins and timing of replanning assessing interfractional cervical and uterine motions three‐dimensionally with daily MR images.

## 2 MATERIALS AND METHODS

### 2.1 Patients and data preparation

For this study, we considered 11 patients with cervical cancer who underwent MR‐guided IMRT using the ViewRay MRIdian (Viewray Inc., Oakwood Village, OH, USA) (Table 1). The pulse sequence used for volumetric imaging was a True Fast Imaging with Steady State Precession sequence. The slice thickness and pixel dimensions of the MR images were 3 mm and 1.5 mm × 1.5 mm, respectively. All patients were asked to urinate and defecate and were then asked to drink 300 mL of water 1 h before entering the treatment room. The daily MR images of each patient were acquired before beam delivery and co‐registered to the planned MR images (reference) based on the bony structure. Thereafter, the uterus, cervix, rectum, and bladder were manually delineated by a single radiation oncologist. The contours were converted to mesh file formats using a commercially available system (ITEM Viewer Planning and Assistant System; ITEM Corporation, Osaka, Japan). Each mesh comprised a unique number of vertices and meshes. Therefore, the surfaces of all acquired meshes were resampled such that we acquired 400 vertices and 796 triangular meshes. As a result, we obtained a point‐to‐point correspondence. The details have been described elsewhere.^21^, ^22^ This study was approved by the institutional review board (approval number: 2020‐556).

**TABLE 1:** Patient characteristics.

Pt^#^	Age (y.o.)	Pathology	TNM	Stage	Chemo	Dose (Gy/fr)	Cx (cm^3^)^a1^	Ut (cm^3^)^a1^
1	63	Ad	T3bN1M0	IIIB	–	46/25	15.9	102.1
2	35	SCC	T3bN1M0	IIIB	CDDP	45/25	103.8	77.9
3	65	SCC	T1b1N0M0	IB	–	45/25	9.9	3.6
4	73	SCC	T3bN1M0	IIIB	–	46/23	15.5	27.2
5	76	SCC	T3bN1M0	IIIB	CDDP	45/25	103.5	78.9
6	67	SCC	T2bN1M0	IIB	CDDP	45/25	38.8	16.9
7	71	SCC	T2bN1M0	IIB	CBDCA	45/25	29.6	33.4
8	70	SCC	T2bN0M0	IIB	CDDP	45/25	20.3	13.6
9	47	SCC	T3bN1M0	IIIB	CDDP	45/25	46.9	82.9
10	88	SCC	T1b1N0M0	IB	–	45/25	13.7	21.7
11	44	SCC	T3bNxM0	IIIB	CBDCA	45/25	45.0	91.2

Abbreviations: Ad, adenocarcinoma; CBDCA, carboplatin; CDDP, cisplatin; Chemo, chemotherapy; Cx, cervix; Pt#, patients; SCC, squamous cell carcinoma; Ut, uterus.

^a^1: Volume at treatment planning (cm^3^).

### 2.2 Calculation of displacement

**FIGURE 1:**
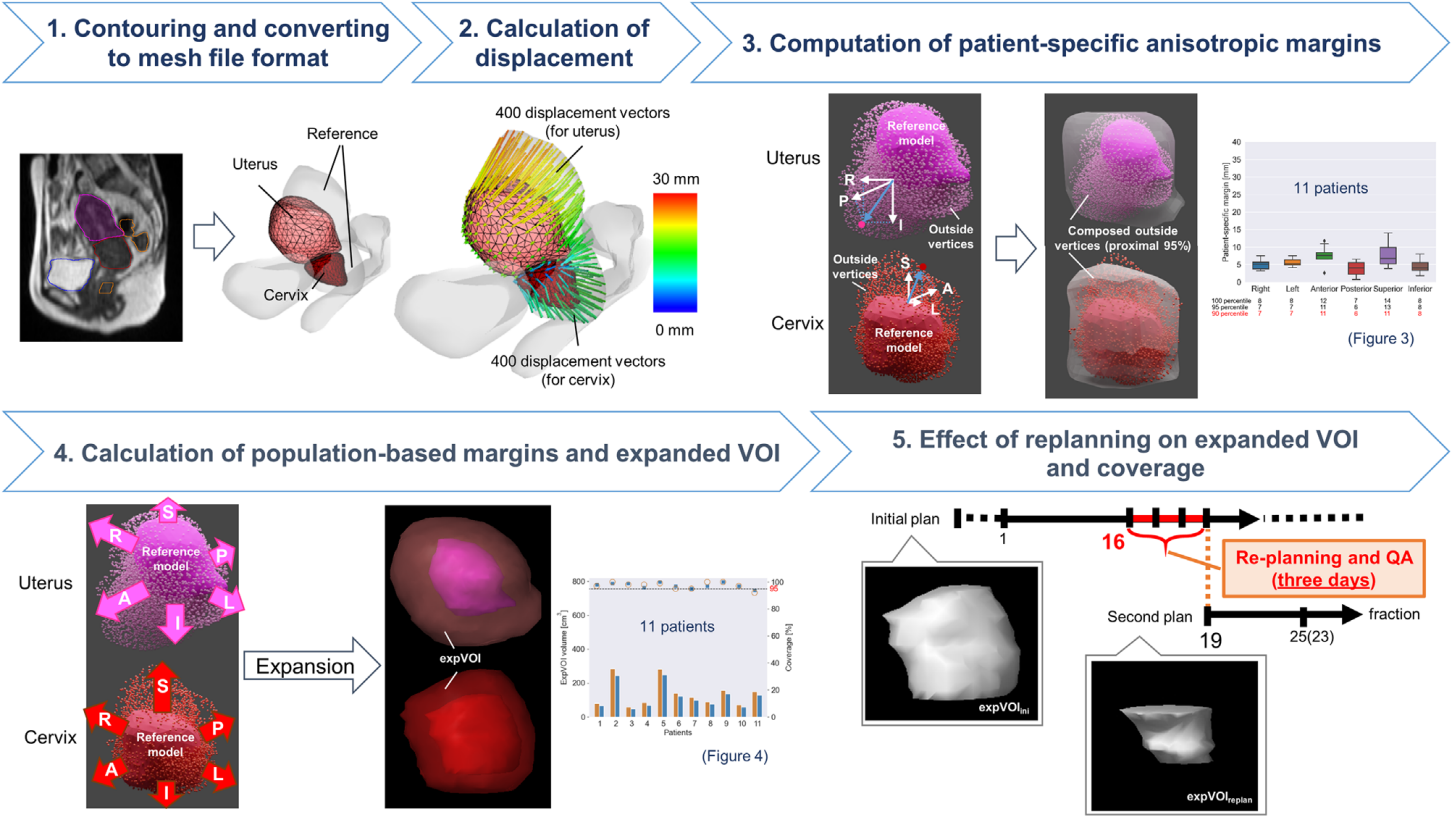
Schematic flow of this study.

Figure 1 shows the flowchart of this study. The daily displacements of the cervix and uterus were acquired as follows: 400 displacement vectors of each corresponding vertex were obtained between each daily shape model and the reference model. The mean displacement was computed by averaging over 400 displacement vectors, defined as the displacement of the day. The cervical volume change was calculated as the percentage of daily cervical volume compared to the reference cervical volume to acquire the trend of cervical volume change. Additionally, Pearson's correlation coefficients were calculated between the cervical volume change and displacement of the cervix or uterus.

### 2.3 Computation of patient‐specific anisotropic margins

The anisotropic margin was computed from the vertices of the daily shape models displaced outside the surface of the reference model (outside vertices). First, the origin was set at the centroid of the reference model, and the outside vertices (1^st^−23^rd^ or 25^th^) were identified. Subsequently, a vector drawn from the surface of the reference model to the vertex was obtained for each outside vertex. Then, the vectors were decomposed along six directions around the origin: right (R), left (L), anterior (A), posterior (P), superior (S), and inferior (I). Lastly, we computed the patient‐specific margins covering the proximal 95% of the outside vertices for all 11 patients based on the lengths of the decomposed vectors in each direction.

### 2.4 Calculation of population‐based margins and expanded VOI

The 90^th^ percentile values of the patient‐specific margins were defined as the population‐based margins in each direction. First, the expanded volume of interest (expVOI) was generated by expanding the reference model depending on the population‐based margins in each direction, which was regarded as a surrogate for a planning target volume. Next, the volume and coverage probabilities of the expVOI were calculated for all patients. The coverage was calculated as the ratio of the vertices of the daily shape models located within the expVOI to the outside vertices. For comparison, expVOI_conv_ was generated by adding conventional margins to the reference cervix and uterus, and the volume of expVOI_conv_ and the coverage probabilities were calculated. Conventional margins (R, L, A, P, S, I) of (5, 5, 15, 15, 10, 10) and (10, 10, 20, 20, 15, 15) mm were used for the cervix and uterus, respectively. The clinical target volume (CTV) margins suggested by Khan et al.^12^ were used as conventional margins for the uterus and margins 5 mm smaller than conventional margins for the uterus were used as conventional margins for the cervix.

### 2.5 Effect of replanning on expanded volume and coverage

A replanning scenario was developed based on the trend in cervical volume change. When the median cervical volume fell below 50% for the first time, the date was set as the new reference date (X^th^ fraction). The replan was simulated based on the shape of the cervix and uterus in the X^th^ fraction. Considering the time required for optimization and quality assurance, the second plan was assumed to start at the (X + 3)^th^ fraction.

Two patient‐specific margins were computed before and after replanning. Before replanning, patient‐specific margins were computed to cover 95% of the outside vertices (1^st^—[X + 2]^th^), whereas after replanning, they were computed to cover 95% of the outside vertices ([X + 3]^th^—23^rd^ or 25^th^). Population‐based margins were determined as the 90^th^ percentile values of each patient‐specific margin. Two expVOIs (expVOI_ini_ and expVOI_replan_) optimized for each population‐based margin were generated before and after replanning. In addition, volume and coverage probabilities were also calculated.

### 2.6 Statistical analysis

Pearson's correlation analysis was performed to study the correlation between cervical volume change and the daily mean displacement of the cervix or uterus. A paired *t*‐test was used to analyze the statistical difference in the margin before and after replanning, as well as the volume and coverage between expVOI_conv_ and expVOI or between expVOI_ini_ and expVOI_replan_. The significance level was set at *p* < 0.05.

## 3 RESULTS

**FIGURE 2:**
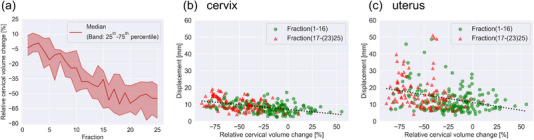
Volumetric data. (a) The trend of relative cervical volume change. Relative cervical volume change and displacement of the (b) cervix and (c) uterus. The solid lines denote regression lines.

Figure 2a shows the median and interquartile range of the cervical volume trend for 11 patients. The median cervical volume fell below 50% for the first time at the 16^th^ fraction. Figure 2b,c shows the cervical volume change and displacement of the cervix and uterus, respectively. Pearson's correlation coefficients between the cervical volume change and the displacement of the cervix or uterus were −0.48 and −0.32, respectively. After the 16^th^ fraction, the cervical volume was smaller than the reference volume, except for one fraction of one patent. The correlations between the bladder or rectal volume change and displacement of the cervix or uterus are shown in Supplementary Materials.

**FIGURE 3:**
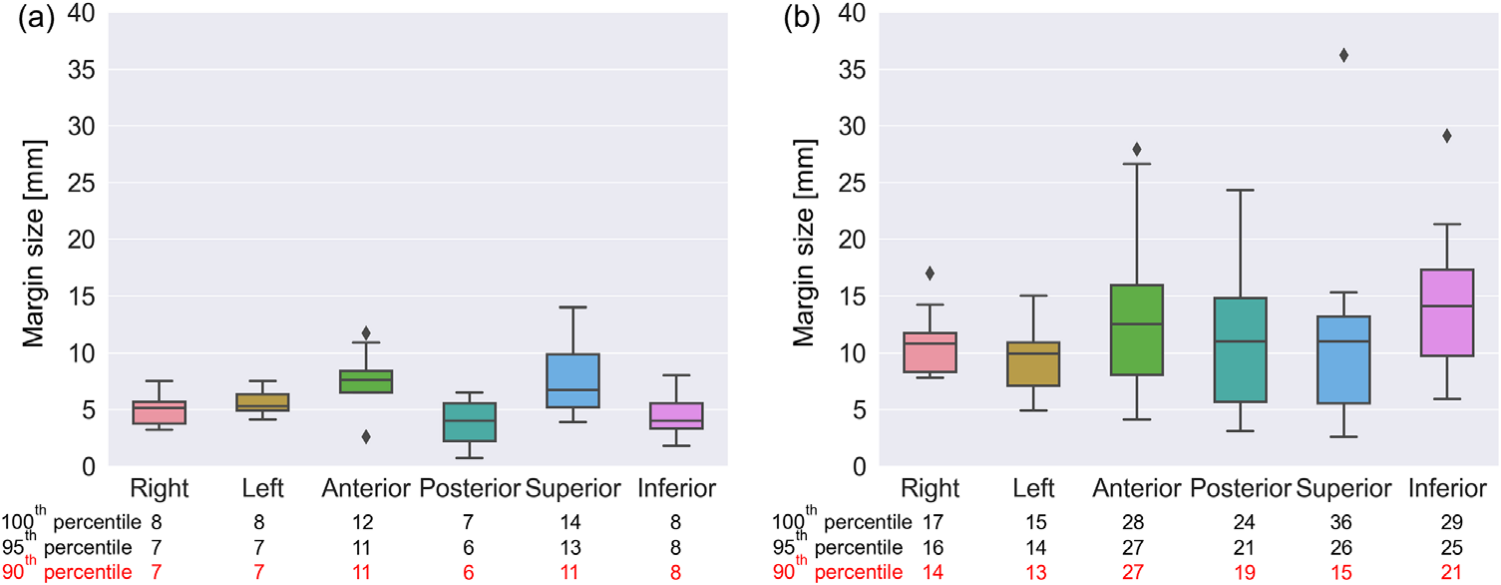
Margin size in the patient group for the (a) cervix and (b) uterus. 90^th^, 95^th^, and 100^th^ percentile values are shown at the bottom of the figure.

Figure 3 shows margin sizes in the patient group. Statistical values, such as the 90^th^, 95^th^, and 100^th^ percentile values, are shown under the legends of R, L, A, P, S, and I. 90^th^ percentile values of patient‐specific margins were used as the population‐based margins. The population‐based margins in (R, L, A, P, S, I) were (7, 7, 11, 6, 11, 8), and (14, 13, 27, 19, 15, 21) mm for the cervix and uterus, respectively.

**FIGURE 4:**
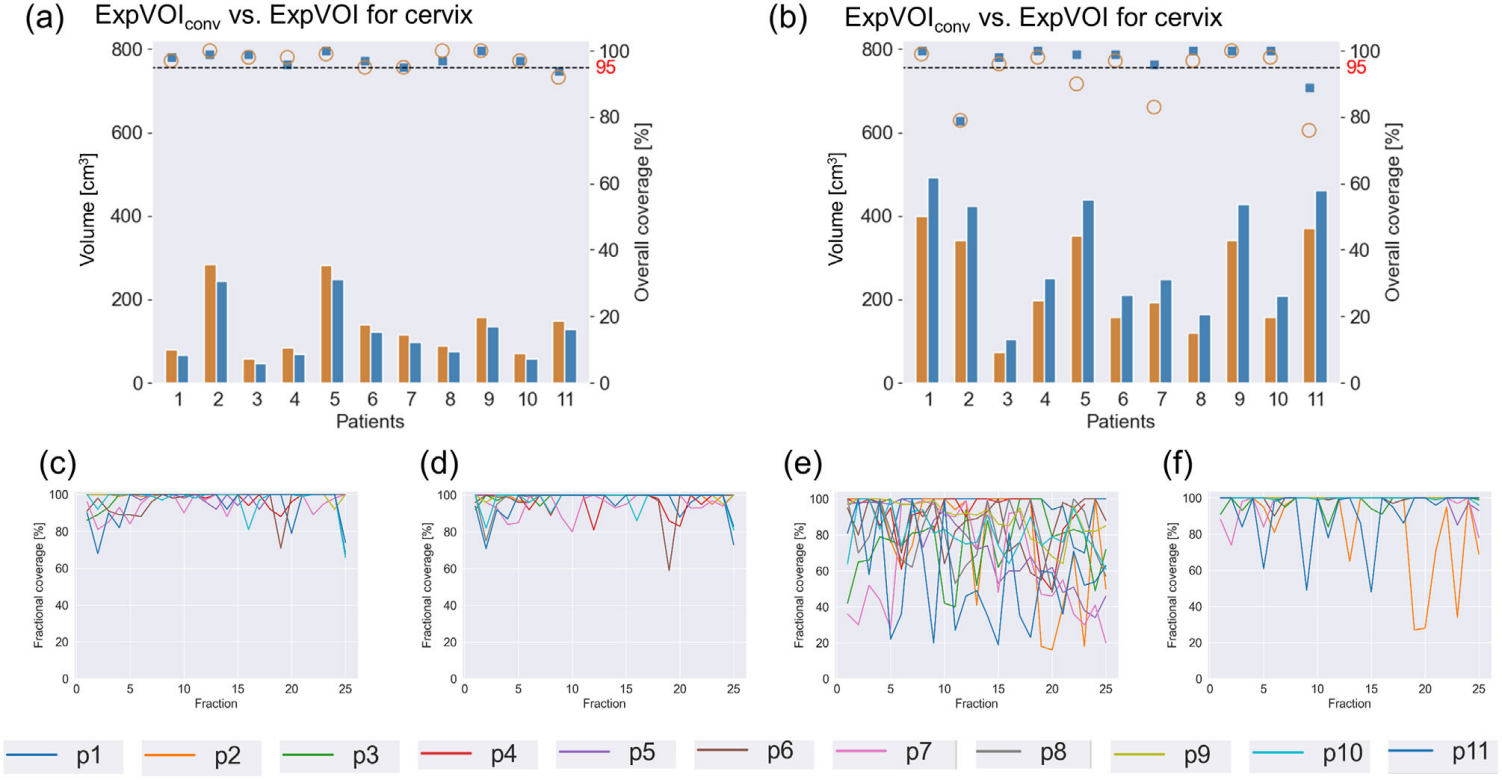
Each patient's volume and overall coverage of the expanded volume of interest (expVOI) and expVOI generated by adding conventional margins (expVOI_conv_) for the (a) cervix and (b) uterus. The volumes of expVOI_conv_ and expVOI are denoted by orange and blue bars, respectively, depending on the left axis. Coverage values of expVOI_conv_ and expVOI are denoted by orange circles and blue squares, respectively, depending on the right axis. The 95% coverage is denoted by a dashed line. Fractional coverage values of expVOI_conv_ and expVOI for the (c,d) cervix and (e,f) uterus are shown for all patients.

Figure 4a,b summarize each individual patient's volume and overall coverage for expVOI_conv_ and expVOI, respectively. For the cervix, the median volumes of expVOI_conv_ and expVOI were 116.4 (range, 56.8−284.9) and 96.9 (range, 47.6−248.8) cm^3^ (*p* < 0.05), respectively, whereas those of the overall coverage of expVOI_conv_ and expVOI were 98 (range, 92−100) and 97 (range, 94−100) % (*p* = 0.85), respectively. For the uterus, the median values of the volumes of expVOI_conv_ and expVOI were 197.6 (range, 72.7−398.7) and 251.7 (range, 104.6−491.5) cm^3^ (*p* < 0.05), whereas those of the overall coverage of expVOI_conv_ and expVOI were 97 (range, 76−100) and 99 (range, 79−100) % (*p* < 0.05), respectively. Figure 4c–f shows the fractional coverage of expVOI_conv_ and expVOI, respectively. The median values of the fractional coverage of expVOI_conv_ and expVOI were 100 (range, 66−100) and 100 (range, 59−100) % (*p* = 0.65) for the cervix, respectively. These values were 85 (range, 16−100) [%] and 100 (range, 27−100) [%] (*p* < 0.05) for the uterus, respectively.

The MR images acquired at the 16^th^ fraction, where the median cervical volume fell below 50% for the first time (Figure 2a), were used as the reference for the second plan, and replanning was performed at the 19^th^ fraction.

**FIGURE 5:**
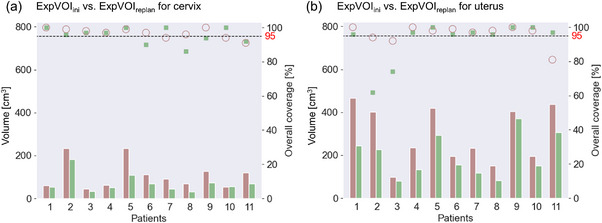
The volume and overall coverage of the expanded volume of interest by adding for each population‐based margin before (expVOI_ini_) and after replanning (expVOI_replan_) for the (a) cervix and (b) uterus. The volumes of expVOI_ini_ and expVOI_replan_ are denoted by brown and green bars, respectively, depending on the left axis. The coverage values of expVOI_ini_ and expVOI_replan_ are denoted by brown circles and green squares, respectively, depending on the right axis. The 95% coverage is denoted by a dashed line.

For the cervix, the population‐based margins in (R, L, A, P, S, I) were (7, 6, 11, 6, 10, 6) and (9, 9, 11, 7, 9, 6) mm, whereas those for the uterus were (16, 12, 28, 15, 16, 17) and (13, 11, 17, 18, 13, 11) mm at the initial planning and replanning stages, respectively. No statistically significant difference was observed in the margin size between the initial and replanning stages (*p* = 0.11 for the cervix and 0.06 for the uterus). Figure 5 shows the volume and overall coverage of expVOI_ini_ and expVOI_replan_ in all patients. For the cervix, the median values of the volumes of expVOI_ini_ and expVOI_replan_ were 90.4 (range, 44.1−232.8) and 54.9 (range, 30.0−181.8) cm^3^ (*p* < 0.05), whereas those of the overall coverage of expVOI_ini_ and expVOI_replan_ were 97 (range, 91−100) and 97 (range, 86−100) % (*p* = 0.45), respectively. For the uterus, the median values of the volumes of expVOI_ini_ and expVOI_replan_ were 234.8 (range, 97.5−464.8) and 155.6 (range, 80.4−369.9) cm^3^ (*p* < 0.05), whereas those of the overall coverage of expVOI_ini_ and expVOI_replan_ were 98 (range, 81−100) and 97 (range, 62−100) % (*p* = 0.32), respectively.

## 4 DISCUSSION

We computed daily interfractional cervical and uterine motions individually in 3D space using MR images. Jadon et al. summarized several reports^23^ and revealed that no researchers had conducted a 3D evaluation, and daily individual assessments or high‐contrast images were lacking. Therefore, the advantage of our study is achieving the exact measurement. Next, we assessed the correlation between cervical volume change and the displacement of the cervix or uterus, which showed weak negative correlations. Furthermore, the volume of the cervix was smaller than that at treatment planning after the 16^th^ fraction, except for one fraction of one patient (Figure 2b,c), which was supported by the clinical view and other reports that the volume shrinks as the treatment progresses.^3^, ^5^, ^9^, ^14^ Therefore, we considered that the tumor was softened as treatment progressed, thereby facilitating the movement of the cervix and uterus (Figure 2b,c).

Table 2 summarizes the margins suggested by previous studies. We found that the margins suggested by other reports were based on a weekly assessment, lower contrast than MR images, or a 2D basis.^7^, ^8^, ^9^, ^10^, ^11^, ^12^ Several investigators did not suggest margins in six directions as they were not suitable for assessing complex variations.^8^, ^10^, ^11^ Our margin for the cervix was smaller than the cervical or gross tumor volume (GTV) margins suggested by Collen et al. or van de Bunt et al. in all directions except for the S‐I direction.^7^, ^9^ Compared to conventional margins, our margin was 2−9 [mm] smaller in the A, P, and I directions. Conversely, our margin for the uterus was equal to or larger than the conventional margins and uterine or CTV margins reported by Collen et al., van de Bunt et al., and Khan et al., except for the margins in the L, P, and S directions.^7^, ^9^, ^12^ Because their 2D basis analysis with weekly or low‐contrast images could not independently evaluate cervical and uterine variations, their variations might interfere with each other, resulting in over‐ and/or underestimation.

**TABLE 2:** Comparison of the measurement method and margin reported by other studies and our study.

Authors	Number of patients	Imaging frequency	Measurement		Target	Margin [mm]		
			Modality	Assessment		R‐L	A‐P	S‐I
Collen et al.^7^	10	Daily	MVCT	2D	Cervix	8–9	17–12	15–9
					Uterus	13–13	19–19	20–19
Chan et al.^8^	20	Weekly	MRI	2D	Cervical os	10–15 (all directions)		
					Uterine funds	10–40 (all directions)		
					Uterine canal	10–12.5 (all directions)		
van de Bunt et al.^9^	20	Weekly	MRI	2D	GTV	12–11	12–14	4–8
					CTV	12–16	24–17	11–8
Wang et al.^10^	8	Biweekly (1, 3, and 5 weeks)	CT	2D	Cervix	9	10	19
					Uterus	14	32	20
Taylor et al.^11^	33	2 days	MRI	2D	CTV	7	15	15
Khan et al.^12^	50	Daily	CBCT	2D	CTV	10–10	20–20	10–10
Our study	11	Daily	MRI	3D	Cervix	7–7	11–6	11–8
					Uterus	14–13	27–19	15–21

Abbreviations: 2D, two‐dimensional; 3D, three‐dimensional; A, anterior; CBCT, cone‐beam computed tomography; CT, computed tomography; CTV, clinical target volume; GTV, gross tumor volume; I, inferior.; L, left; MRI, magnetic resonance imaging; MVCT, megavoltage computed tomography; P, posterior; R, right; S, superior.

Figure 4a,c,d shows that expVOI for the cervix provided comparable coverage compared to expVOI_conv_, whereas the volume of expVOI decreased by 12%−19% in all patients. Alternatively, the coverage of expVOI was significantly higher than that of expVOI_conv_ in the uterus (Figure 4b,e,f). The coverage of expVOI_conv_ decreased by 20% or less for three patients, and the daily variation in coverage was more notable than that in expVOI. For example, the coverage was 100% on one fraction but decreased to 18% on the next fraction for patient 2, as shown in Figure 4e. This indicated that interfractional variations were unpredictable, and hence, daily observation for interfractional movement would be required. We defined the population‐based margins as the 90^th^ percentile values of patient‐specific margins. If 100^th^ percentile values were adopted, the overall coverage and fractional coverage would improve. However, the 100^th^ percentile values for the uterus were (17, 15, 28, 24, 36, 29) in (R, L, A, P, S, I) [mm], which would be too large for application in clinical practice. Therefore, our margins and coverage were clinically realistic.

In this study, the timing of replanning was universally picked at the 16^th^ fraction (approximately 30 Gy) instead of personalized time points based on individual volume shrinkage, which was similar to that reported by several investigators.^5^, ^9^, ^14^ It is crucial to closely monitor each individual volume change and trigger personalized adaptive planning decisions; however, this is beyond the scope of this study. Interestingly, although the volume of expVOI_replan_ was smaller than that of expVOI_ini_ for both the cervix and uterus, no significant differences were observed between the coverage of expVOI_ini_ and expVOI_replan_. The median volume reduction of expVOI_replan_ was 37 (range, 11−56) and 30 (range, 8−50) % for the cervix and uterus at the 16^th^ fraction, respectively. Nevertheless, the margin size should not be reduced for the cervix and uterus. As shown in Figure 2b,c, the displacement of the cervix and uterus increased as the treatment progressed. Therefore, it is likely that a margin equivalent to that at the initial planning was required for replanning.

Nonetheless, this study has some limitations. First, delineation was conducted by a single radiation oncologist on the MR images with fixed imaging protocols. As several investigators indicated,^24^, ^25^ these factors may cause the results to change. Second, the margins and coverage were calculated based on the same patient group. Initially, margins should have been applied to a different patient group for the coverage calculation; however, it was impossible owing to the small number of cases, although a total of 273 datasets were used. Additionally, it was challenging to decide a particular confidence level and an appropriate number of patients for margin calculation. As shown in Table 2, while other studies considered various numbers of patients, none of them defined the confidence level and appropriate number of patients. Therefore, 90^th^ percentile values of patient‐specific margins were defined as population‐based margins instead of confidence level. Lastly, because the intrafractional variations were not assessed, it is unclear whether the margins determined in this study compensated for intrafractional variations.^26^ Intrafractional variations can also be assessed with delineated organ data acquired during beam delivery.

## 5 CONCLUSION

We determined the margins and the timing of replanning through detailed daily analysis using MR images. The margins of the cervix were smaller than conventional margins and the margins suggested by other studies in some direction, while the margins of the uterus were larger in almost all directions. The timing of replanning was determined to be the 16^th^ fraction, and the volume of expVOI_replan_ decreased. However, the margin equivalent to that at the initial planning was required for replanning.

## AUTHOR CONTRIBUTIONS

Yukako Kishigami and Mitsuhiro Nakamura planned the study. Yukako Kishigami performed the statistical analysis and drafted the manuscript. Yukako Kishigami, Mitsuhiro Nakamura, Megumi Nakao, and Hiroyuki Okamoto conceived the study and participated in its design and coordination. Megumi Nakao, Hiroyuki Okamoto, Ayaka Takahashi, and Hiroshi Igaki helped draft the manuscript. All authors read and approved the final manuscript.

## CONFLICT OF INTEREST STATEMENT

The authors declare no conflicts of interest.

## Supporting information

Supplementary Information

Supplementary Information

Supplementary Information

Supplementary Information

## Data Availability

The data that support the findings of this study are not available.
