# Supplementary figures and images for: Three‐dimensional assessment of interfractional cervical and uterine motions using daily magnetic resonance images to determine margins and timing of replanning
Source: J Appl Clin Med Phys. 2023 Jun 15;24(10):e14073. doi: 10.1002/acm2.14073 (PMC10562032; doi:10.1002/acm2.14073)

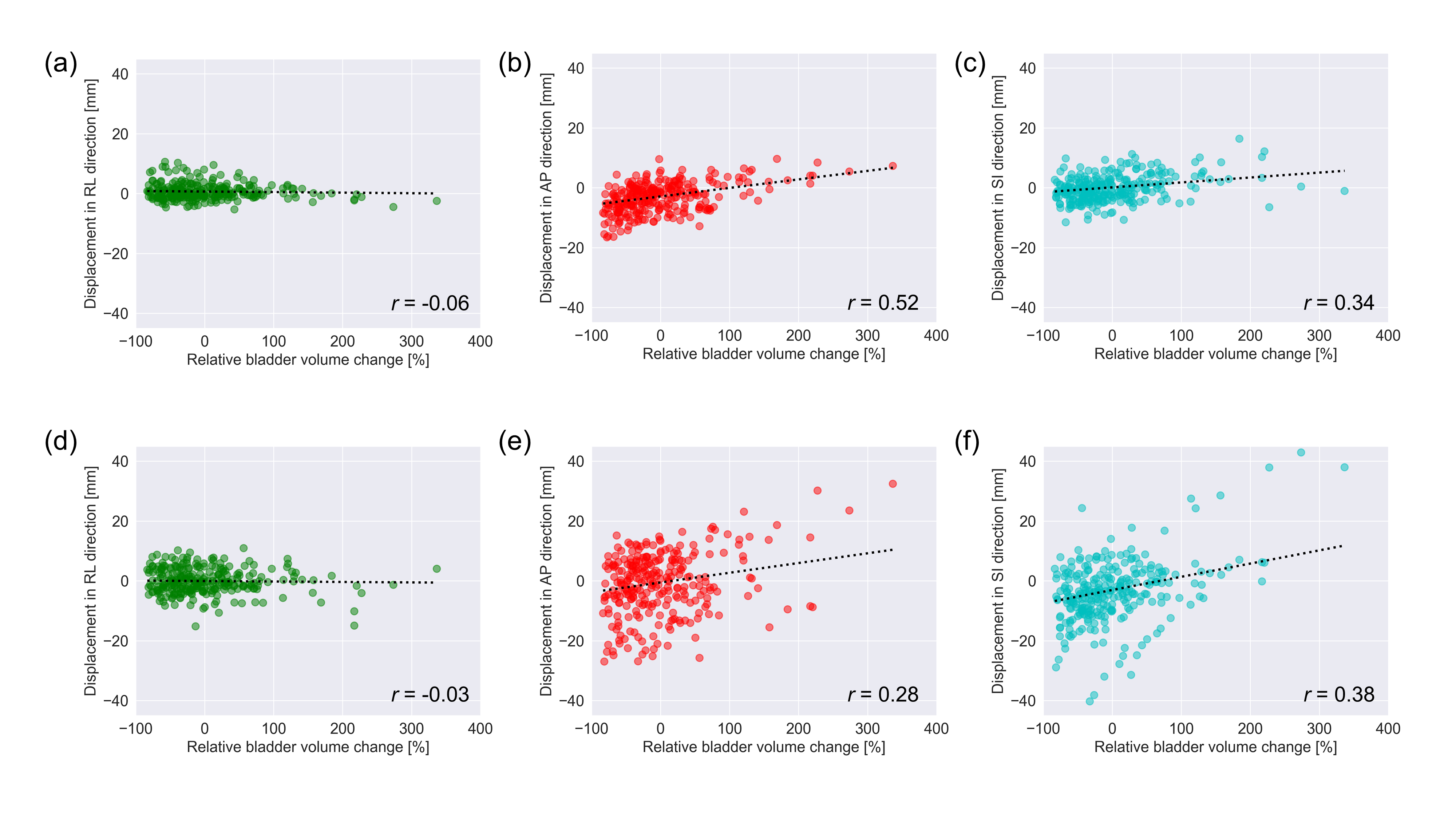

Supplement: Supplementary file 1 — Supplementary Information [file ACM2-24-e14073-s003.tif]

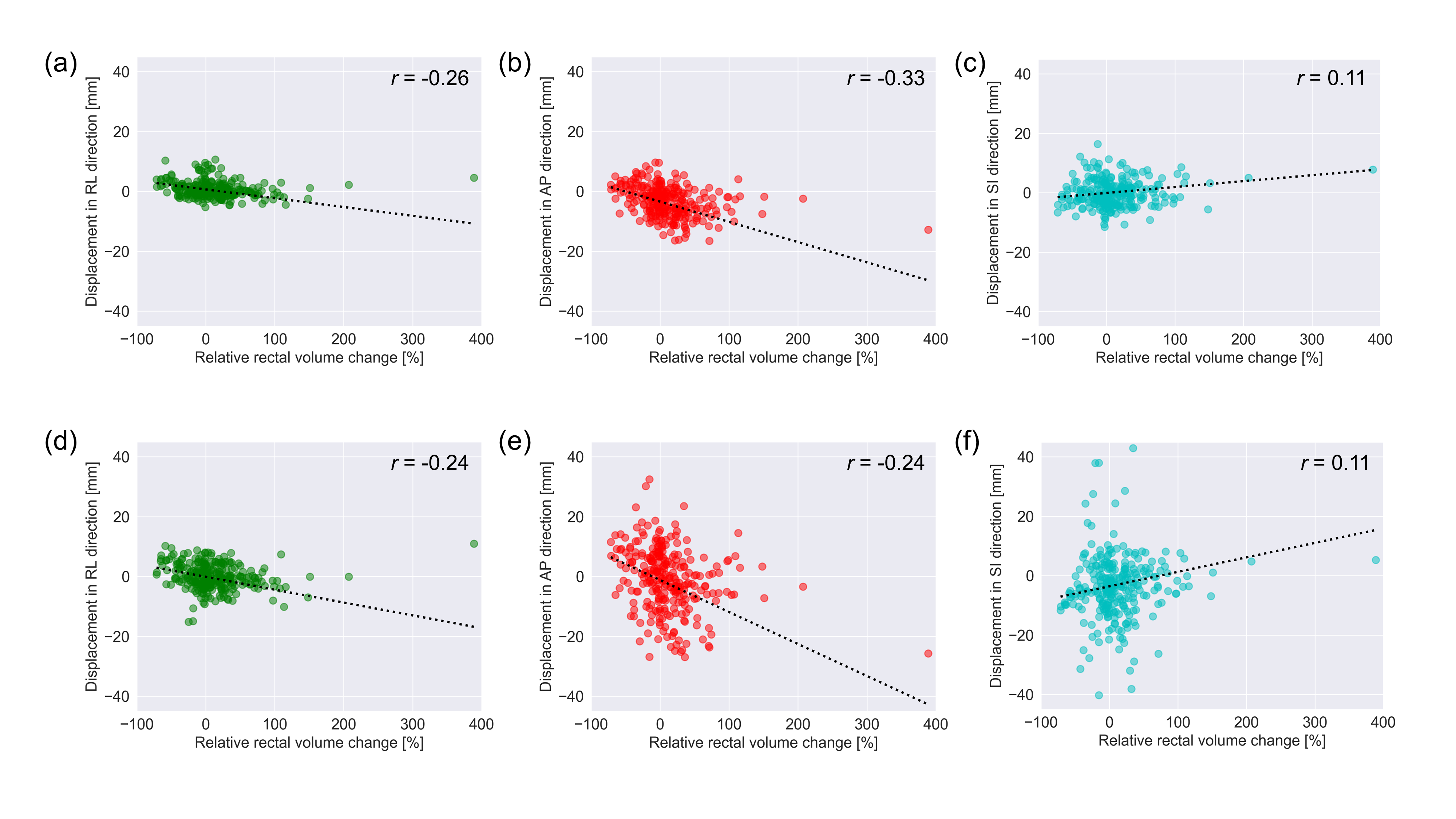

Supplement: Supplementary file 2 — Supplementary Information [file ACM2-24-e14073-s001.tif]
