# Supplementary material for: Three‐dimensional assessment of interfractional cervical and uterine motions using daily magnetic resonance images to determine margins and timing of replanning
Source: J Appl Clin Med Phys. 2023 Jun 15;24(10):e14073. doi: 10.1002/acm2.14073 (PMC10562032; doi:10.1002/acm2.14073)
Supplement: Supplementary file 4 — Supplementary Information [file ACM2-24-e14073-s004.pdf]

Supplementary materials

Materials and methods

Correlation with the volume change of rectum or bladder

Pearson's correlation analysis was performed to assess the correlation between the bladder or rectal volume change and displacement of the cervix or uterus. The bladder or rectal volume change was calculated as the percentage of daily bladder or rectal volume compared to the reference bladder or rectal volume.

Result

Correlation between OAR volume and target displacement

For both the cervix and uterus, bladder volume was correlated with the mean displacement of the AP (Pearson's correlation coefficients for the cervix or uterus: $r = 0.52$ or 0.28) and SI ($r = 0.34$ or 0.38) (**Supplementary Figure 1)**, and rectal volume was correlated with a mean displacement of RL ($r = 0.26$ or 0.24) and AP ($r = 0.32$ or 0.24) in **Supplementary Figure 2**.

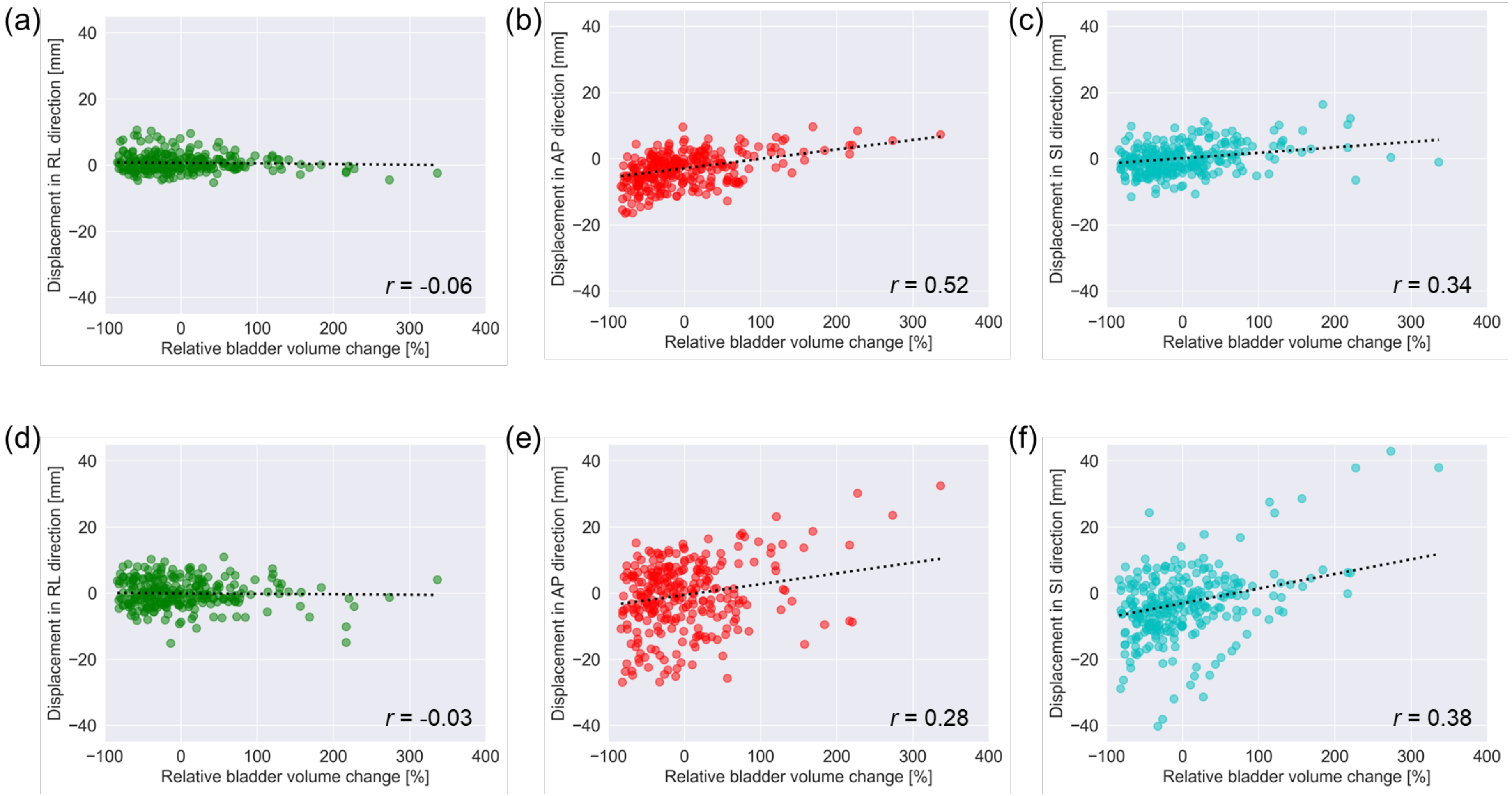

Supplementary Figure 1. Relationship between relative bladder volume change and displacement of cervix (a, b, c) or uterus (d, e, f) in the RL (a, d), AP (b, e), and SI (c, f) directions. The solid lines denote the regression lines.

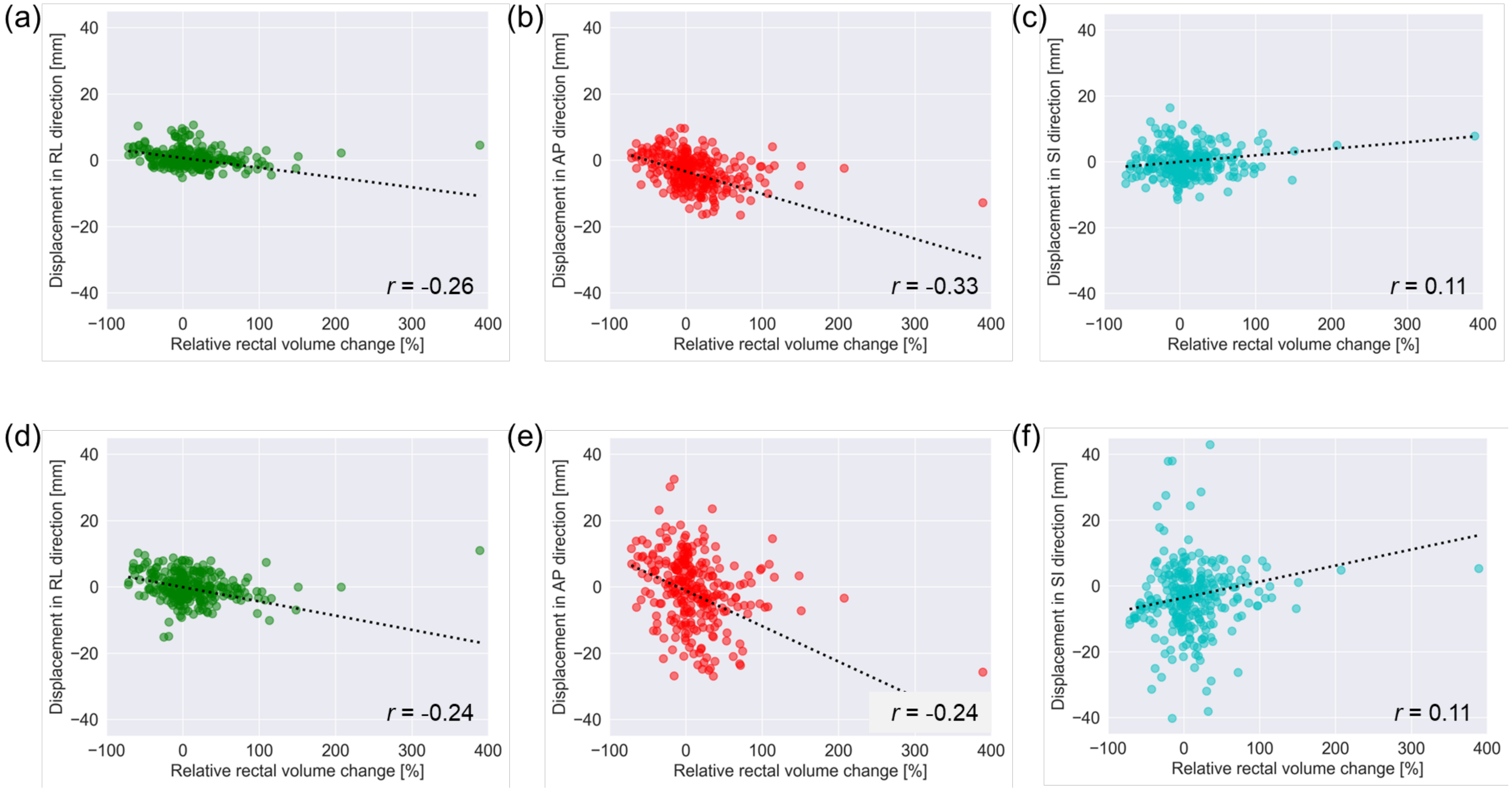

Supplementary Figure 2. Relationship between the relative rectal volume change and displacement of the cervix (a, b, c) or uterus (d, e, f) in the RL (a, d), AP (b, e), and SI (c, f) directions. The solid lines denote regression lines.
